# Using working memory performance to predict mathematics performance 2 years on

**DOI:** 10.1007/s00426-020-01382-5

**Published:** 2020-07-10

**Authors:** Katie Allen, David Giofrè, Steve Higgins, John Adams

**Affiliations:** 1 School of Education, University of Durham, Durham, UK; 2 Dipartimento di Scienze della Formazione (DISFOR), University of Genoa, Genoa, Italy; 3 Department of Psychology, University of Durham, Durham, UK

## Abstract

A number of previous studies have used working memory components to predict mathematical performance in a variety of ways; however, there is no consideration of the contributions of the subcomponents of visuospatial working memory to this prediction. In this paper we conducted a 2-year follow-up to the data presented in Allen et al. (Q J Exp Psychol 73(2):239–248, 2020b) to ascertain how these subcomponents of visuospatial working memory related to later mathematical performance. 159 children (*M* age = 115.48 months) completed the maths test for this second wave of the study. Results show a shift from spatial–simultaneous influence to spatial–sequential influence, whilst verbal involvement remained relatively stable. Results are discussed in terms of their potential for education and future research.

## Introduction

Using working memory to predict mathematical attainment is an area of study that has gained a significant amount of traction in recent years. Mathematics is a broad field and there has been extensive research across a number of aspects of mathematics and working memory which has been summarised in reviews and meta-analyses, from studies of typically developing populations (Friso-van den Bos et al., 2013; Raghubar et al., 2010), to the relationship with learning difficulties in mathematics generally (David, 2012; Swanson & Jerman, 2006) and in terms of the verbal and numerical domains in particular (Peng & Fuchs, 2016). According to the multicomponent model (Baddeley & Hitch, 1974), working memory involves subcomponents relating to the processing of visuospatial and phonological stimuli. The components of working memory have been reliably linked to academic performance on a number of occasions (e.g., Alloway & Passolunghi, 2011; Holmes & Adams, 2006; Van de Weijer-Bergsma, Kroesbergen, & Van Luit, 2015; see Peng, Namkung, Barnes, & Sun, 2016 for a review of this literature) with a reasonable amount of evidence suggesting visuospatial working memory is more influential in younger children (e.g., Caviola, Mammarella, Lucangeli, & Cornoldi, 2014; Clearman, Klinger, & Szucs, 2017; Holmes, Adams, & Hamilton, 2008). There is also a smaller, though not insignificant, amount of evidence indicating the involvement of verbal working memory (e.g., Kyttälä, Kanerva, Munter, & Björn, 2019; Wilson & Swanson, 2001); a finding we replicated at time 1 (T1) of this study (Allen, Giofrè, Higgins & Adams, 2020b).

At T1, results revealed that, when compared directly to spatial–simultaneous and spatial–sequential measures, verbal-numeric tasks were more predictive of mathematics in 7–8-year-old children. Similarly, Allen, Giofrè, Higgins and Adams (2020a) demonstrated that verbal working memory (non-numeric) was more predictive of mathematical performance in younger children, with a move toward visuospatial influence in older children. It is not yet fully understood, however, how these components relate specifically to mathematical attainment on a longitudinal basis. There is some evidence suggesting visuospatial working memory is influential in the prediction of mathematics over a number of years (e.g., Bull, Espy, & Wiebe, 2008; De Smedt et al., 2009; Fanari, Meloni, & Massidda, 2019; Geary, 2011; Hilbert, Bruckmaier, Binder, Krauss, & Bühner, 2019; Li & Geary, 2017); however, as indicated by Hilbert et al. (2019), it is necessary to consider the mathematics test used for the purposes of these studies. In some cases, standardised measures, in line with the curriculum of the country are used, in which case more credence can be given to the real-life applicability of the findings. However, oftentimes researchers use tests designed specifically for the purposes of their study, in which case they lack the necessary real-world application of the findings. There are also findings to the contrary indicating the importance of verbal working memory (e.g., Geary, Nicholas, Li, & Sun, 2017; Kyttälä et al., 2019), the varying influence of the subcomponents depending on the area of mathematics in question (van der Ven et al., 2013), and even that working memory is not directly predictive of mathematics (Gathercole et al., 2003), especially when other precursor measures of mathematics are included (Krajewski & Schneider, 2009). One area that these studies do not account for is the format of the testing in each of the domains of working memory, for example, visuospatial stimuli can be shown both simultaneously and sequentially, which may have an influence on their predictive value, particularly when considering different areas and levels of mathematics.

There is growing evidence for the subdivision of visuospatial working memory into spatial–simultaneous and spatial–sequential categories, based on the presentation of the information during the encoding phase (e.g., as in Blalock & Clegg, 2010; Lanfranchi, Carretti, Spanò, & Cornoldi, 2009). Spatial–simultaneous tasks require participants to recall a visual array when all items are presented simultaneously, whilst spatial–sequential tasks require recall of visual locations presented sequentially, generally in a given order (e.g., Mammarella et al., 2006; Mammarella, Pazzaglia, & Cornoldi, 2008). Evidence for a double dissociation between the two subtypes of visuospatial working memory (Mammarella et al., 2006, 2018; Wansard et al., 2015) presents the possibility that deficits in these subcomponents act as a specific vulnerability for mathematical difficulties. This is particularly pertinent if there is evidence of a longitudinal predictive relationship between the subcomponents and mathematics. The relationships between the subcomponent of visuospatial working memory and mathematics are not, as yet, thoroughly understood, therefore, this paper aims to contribute to this understanding to develop our ability to predict mathematics performance from working memory capacity.

There are a number of issues associated with the selection of a measure of mathematics for research purposes, including, but not limited to, the applicable age range, the standardisation procedure for the test, and design purely for research purposes, all of which increase the risk of a lack of reliability and validity of the measure in a classroom setting. To bypass some of these issues, a standardised measure was chosen which was suitable for an appropriate age range, which was standardised on a UK sample, and which was designed to map directly on to the current National Curriculum for England and Wales. Mapping onto the National Curriculum means that all children involved in the study have been exposed to the same mathematical content, therefore, should have similar background experience in terms of answering the questions. The same mathematics test was used as at T1 (Access Mathematics Test). This test was selected as it covered topics appropriate for children aged 6-12, therefore, the same measure could be administered at both time points to make a direct comparison. The test has two forms, A and B, which are designed to be equal to each other in terms of both difficulty and the distribution of topics assessed (see Access Mathematics Test Handbook for this information). At T2, the alternate form was administered to that which the children had done at T1 (if form A was used at T1, form B was used at T2, and vice versa) such that children had not had previous exposure to the same questions so that their performance was not skewed in any way.

This study aims to identify whether there is a relationship between working memory measures taken in year 3 and a mathematics measure taken in year 5, and if so, whether the nature of this relationship is the same as when the mathematics measure was also taken in year 3. We aim to identify which working memory predictors can predict mathematical performance in year 5 when mathematical performance in year 3 is taken into account. We expect to see a shift in the extent of the relative contributions of the elements of working memory, particularly between the verbal and visuospatial elements given the suggestion of a developmental shift between the two ages the children were tested at.

## Method

### Participants

The initial sample included 214 7–8-year-old children, however, subject attrition over the 2-year period resulted in a final sample of 159 9–10-year-old children (76 male and 83 female, *M* age = 115.48 months, SD = 3.43). We strove to re-test as many of the original opportunity sample of children, now in year 5, as possible. Opt-out parental consent was obtained, as with the first administration of the study, to reduce bias in the sample (Krousel-Wood et al., 2006). The study was approved by the School of Education Ethics Committee at the University of Durham. Children with special educational needs, intellectual disabilities, or neurological and genetic conditions were not included in the study. Those who did not complete the first administration phase of the study were not included in the analysis, such as children who had entered the school within the last 2 years.

### Design and procedure

Previously, children had been tested individually on working memory measures (spatial–simultaneous, spatial–sequential, and verbal) and mathematics as a class group in year 3 (see Allen et al., 2020b for a full description of this phase) to form Time 1 of the study. This second phase (Time 2) of the study required only a mathematics test, therefore, children were tested as a class group. Working memory measures were not administered at this stage as the intention was to understand whether it is possible to design a measure to be administered at the beginning of formal schooling to predict whether a child is likely to encounter mathematics difficulties in the future, hence this would only be measured once. Testing was done in the child’s usual classroom and with their class teacher present to minimise stress, but was completed under typical test conditions. The test was administered according to the instructions in the testing manual (see below for further explanation), with a 10-min warning prior to the end of the test. Paper and pencil format was used and children could request a question be read aloud to account for those children with a lower reading ability. No further help was given as part of the reading process, nor were any numbers that may have been particularly pertinent to the question, for example “The River Nile is 3256 km long. Round this to the nearest 1000 km.” would be read as “The River Nile is this long (point to number). Round this to the nearest this distance (point to number)”. We used a correlational design to investigate the relationships between earlier working memory measures and current mathematics performance.

### Measures

#### Working memory

Working memory measures from T1 were used for this analysis. At T1, measures of verbal working memory [digit recall, backwards digit recall, and counting recall, as presented in the Working Memory Test Battery for Children; Gathercole & Pickering, 2001)], spatial–simultaneous working memory (4 × 3 and 4 × 4 dot matrices tasks—children were presented grids containing dots and were required to recall the positions of the dots), and spatial–sequential working memory (3 × 3 and 4 × 3 dot matrices tasks—children were presented grids in which dots appeared sequentially and were required to recall the positions of the dots in no specific order—and block recall; Corsi, 1972) were administered to all children prior to the mathematics test. See Allen et al. (2020b) for a full description of the measures taken during phase one.

#### Mathematics

*Access Mathematics Test (AMT)* The AMT is a standardised measure of National Curriculum mathematics, designed to test children aged 6–12 years. It, therefore, provides clear evidence for how well each child performs in individual areas of mathematics, as well as overall. The AMT covers the requirements of the National Curriculum in England and Wales, where children are required to understand number, measurement, geometry, and statistics, hence providing an ecologically valid measure of a child’s school performance. Questions cover number (e.g., “the distance from New York to London is 3457 km. Write this distance to the nearest 1000 kilometres”), operations (e.g., “write the missing number. __ ÷ 5 = 35”), fractions including ratio (e.g., “peanuts cost 40p for 100 g. How much does 120 g of peanuts cost?”), geometry (e.g., “the point A is moved three squares to the right and two squares down. Write the coordinates of this new point A”), measures (e.g., “how many 20p coins are there in £13?”), and statistics (e.g., “this bar chart, from a spreadsheet, shows the number of pets each pupil owns. How many pupils own 2 pets or more?”).

Children were read the instructions set out for the AMT, which included a time limit of 45 min, clarification of where to write their answer on the paper, and an explanation that workings were allowed on the paper, providing their answer was clearly written in the correct space. Typical classroom test conditions were adopted throughout. Children were permitted to request questions be read aloud to them should they have difficulties so as not to disadvantage those with weaker reading abilities; however, no further explanation of the question, or what was required, was given. No discontinuation rule was employed as children were instructed to complete as many questions as they could, but that questions were also included for children much older than they were so not to worry if they could not complete them all. The total number of test items for this test is 60, with a maximum score of 60.

### Data analytic strategy

All analyses were performed using R (R Core Team, 2018). The R program (R Core Team, 2018) with the “lavaan” library (Rosseel, 2012) was used to conduct structural equation modelling (SEM). Model fit was assessed using a variety of indexes according to the criteria suggested by Hu and Bentler (1999). In particular, the Chi-square (*χ*^2^), the comparative fit index (CFI), the non-normed fit index (NNFI), the standardised root mean square residual (SRMR) and the root mean square error of approximation (RMSEA) were used to evaluate model fit, whilst the Akaike information criterion (AIC; the lower the better) and the Chi-square difference (with results not statistically significant favouring more parsimonious models) were used to compare the fit of alternative models.

Full‐information maximum likelihood (FIML) estimation was used to handle missing data in our analyses. This method offers unbiased estimates under missing data patterns such as missing completely at random (MCAR) or missing at random (MAR). The pattern of missingness was tested using correlations (see Kabacoff, 2015 for the rationale). Missing values at T2 were coded 1 for missing and 0 for present. This dummy variable was then correlated with our measures at T1 (i.e., WM and mathematics). None of the correlations were particularly large or striking (*r*s < 0.17), which suggests that the data deviate minimally from MCAR and may be MAR. Therefore, the assumption that data are either MCAR or MAR is justified. Maximum likelihood estimation with robust (Huber-White) standard errors and a scaled test statistic was used for the analyses. This test provides robust estimates and should be preferred in every normal application using SEM (Rosseel, 2010).

The influence of age in months was taken into account by calculating standardised residuals for each variable included in this study. Residuals were calculated by entering each score as the dependent variable and age as predictor (see Allen et al., 2020b; Giofrè & Mammarella, 2014 for a similar method).

## Results

Table 1 shows correlations among variables at T1 and at T2 together with descriptive statistics for these variables.

**Table 1:** Pairwise correlation matrix with raw score correlations below the leading diagonal and age covaried correlations above the diagonal, including means and standard deviations for each measure

	1	2	3	4	5	6	7	8	9	10
1. Simultaneous 4 × 3	–	0.685*	0.484*	0.437*	0.407*	0.352*	0.321*	0.180*	0.410*	0.430*
2. Simultaneous 4 × 4	0.681*	–	0.416*	0.433*	0.407*	0.305*	0.289*	0.122*	0.397*	0.408*
3. Sequential 3 × 3	0.488*	0.415*	–	0.573*	0.343*	0.301*	0.257*	0.112	0.308*	0.414*
4. Sequential 4 × 3	0.440*	0.430*	0.576*	–	0.363*	0.257*	0.277*	0.139*	0.300*	0.372*
5. Block recall	0.416*	0.406*	0.349*	0.368*	–	0.287*	0.239*	0.077	0.242*	0.238*
6. Counting recall	0.358*	0.308*	0.300*	0.253*	0.289*	–	0.444*	0.322*	0.385*	0.420*
7. Backward digit	0.325*	0.290*	0.259*	0.279*	0.243*	0.445*	–	0.325*	0.318*	0.390*
8. Digit recall	0.180*	0.123*	0.110*	0.135*	0.076	0.325*	0.325*	–	0.156*	0.204*
9. Math assessment Y3	0.417*	0.399*	0.310*	0.302*	0.248*	0.390*	0.320*	0.158*	–	0.832*
10. Math assessment Y5	0.420*	0.407*	0.411*	0.369*	0.232*	0.413*	0.387*	0.202*	0.823*	–
M	28.28	20.11	18.7	15.36	21.5	16.33	10.52	26.61	11.72	24.19
SD	5.99	6.85	4.72	4.23	4.09	3.99	3.08	3.51	6.64	10.140

*p < 0.05 one tail

The main aim of this longitudinal paper was to evaluate the impact of working memory on mathematics, controlling for the effects of mathematics at T1. To achieve this aim, SEM was used, fitting a model with three latent variables for working memory (spatial–sequential, spatial–simultaneous, and verbal), and two observed variables for mathematics at T1 and T2. In this model, the three correlated working memory factors were predicting mathematics at T1 and T2, whilst mathematics at T1 was also predicting mathematics at T2. This latter path allows us to control for potential autoregressive effects, i.e., the performance in mathematics at T2 is controlled for by the performance in mathematics at T1. This model design allows us to control for the shared contribution of working memory, i.e., the effect of each working memory factor is over and above the effect of the other predictors.

**Fig. 1:**
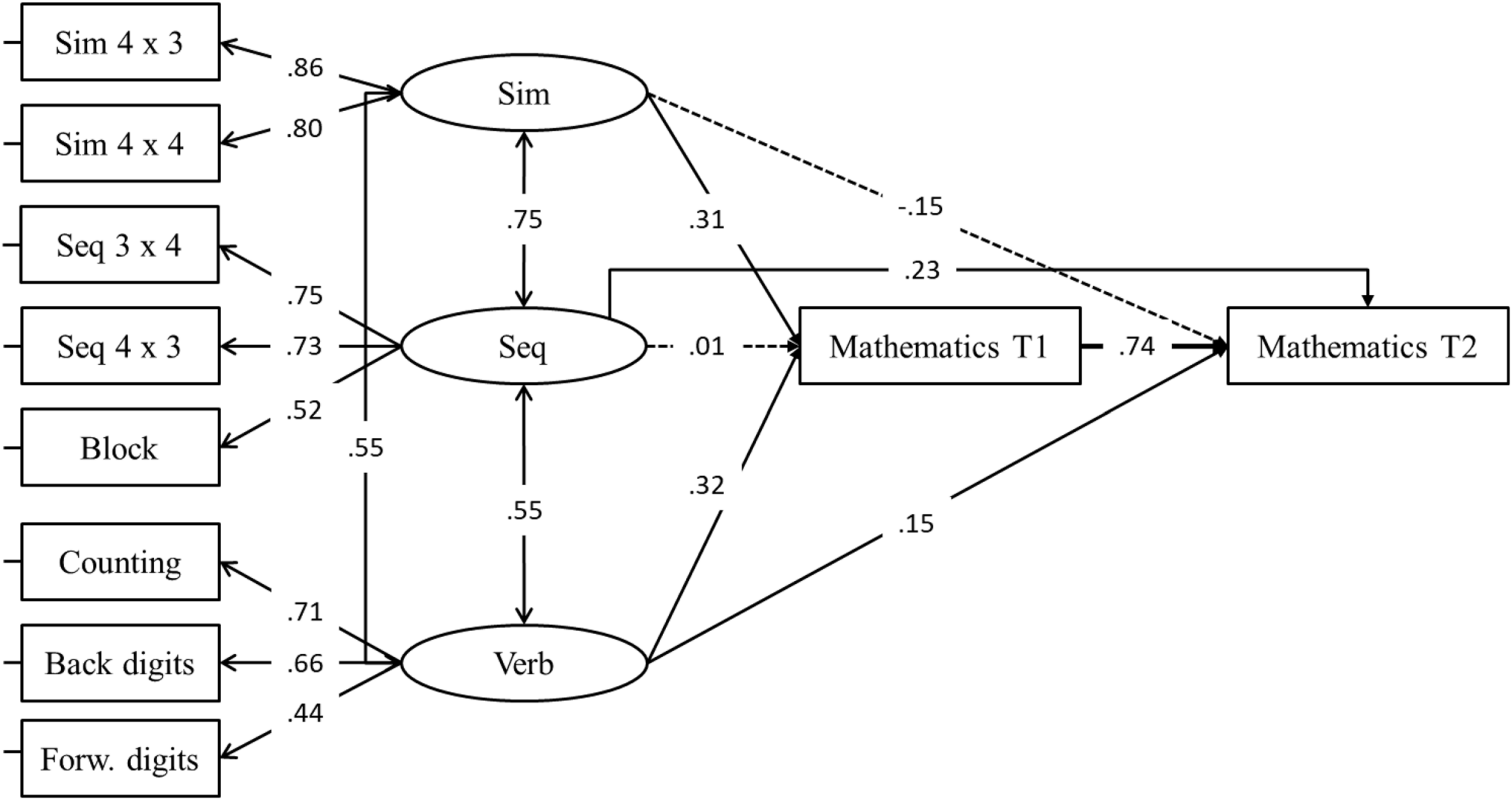
SEM model for working memory, mathematics T1 and T2. Solid lines represent statistically significant paths (*p* < 0.05)

The fit of the model was good, *χ*^2^(27) = 20.73, *p* = 0.799, RMSEA = 0.000, SRMR = 0.029, CFI = 1.00, NNFI = 1.014 (Fig. 1). In this model, paths from simultaneous and verbal working memory factors to mathematics at T1 were statistically significant, whilst the path from sequential working memory was not. As for mathematics at T2, the path from mathematics at T1 as well as paths from sequential and verbal working memory, were statistically significant albeit with a small effect size, whilst the path from simultaneous working memory was not.

### Additional analyses

Mathematics is a broad concept, addressing for example measurement, properties, and relations of quantities (Peng et al., 2016). The test we used to evaluate mathematics includes different components, making it possible to distinguish among them. This comparison is of particular interest, because it can be argued that the relation between verbal, spatial–simultaneous and spatial–sequential working memory can potentially be affected by different types of mathematics skills. It can also be argued that there might be a shift in this relationship due to a change in the curriculum (i.e., different proportions of the different domains). According to this hypothesis, one could assume that verbal-numeric working memory could have a stronger relation to word-problem-solving or to number-based mathematics skills (e.g., calculation), and visuospatial working memory to visual-related mathematics skills (e.g., geometry). To investigate this issue, we performed some additional analyses.

In a fist SEM model, similar to what we did in the aforementioned SEM model, three exogenous working memory factors (i.e., variables that are not caused by another variable in the model) were calculated (i.e., simultaneous, sequential and verbal). These working memory factors were allowed to correlate. As for the endogenous variables (i.e., variables that are caused by one or more variables in the model), rather than including the overall score for mathematics as we did before, all subdomains were included separately (i.e., number, operations, fractions including ratio, geometry, measures, statistics including probability). Residual errors of mathematics domains were also allowed to correlate, this is normal practice in SEM when tasks, as in this case, belong to the same constructs and are intrinsically related in nature, that is they share a significant portion of the variance over and above what is accounted for by working memory factors in this case. In the model, each working memory factor was independently predicting each mathematic variable. In this first model all betas were freely estimated (i.e., were supposed to be independent from each other). The fit of this model was satisfactory, *χ*^2^(47) = 48.70, *p* = 0.405, RMSEA = 0.013, SRMR = 0.029, CFI = 0.998, NNFI = 0.997, AIC = 13,200.

Having established that the model provided a satisfactory fit we tested several alternative nested models in which the betas from working memory to each mathematic domain were constrained to be equal across the tasks (i.e., the relationship to working memory was considered to be similar in each individual mathematic subdomain). We took a multi-step approach, fixing one group of betas at a time. In the first model, betas from simultaneous working memory to each mathematic domain were constrained to be equal (assumed to be similar across each individual math variable). The fit of this model, was similar to the previous model, *χ*^2^(52) = 53.24, *p* = 0.426, RMSEA = 0.011, SRMR = 0.030, CFI = 0.999, NNFI = 0.998, AIC = 13,195. Importantly this latter model had a lower AIC, was more parsimonious (i.e., had a higher number of degrees of freedom), and was not statistically different from the previous one, Δ*χ*^2^(5) = 4.38, *p* = 0.4958, meaning that this model should be preferred over the previous one. This finding indicates that increasing the complexity of the model and assuming different betas (i.e., different relationships) from the simultaneous working memory factor to each mathematic subdomain was not necessary (i.e., the simultaneous working memory factor had a similar impact on each individual mathematic task).

In a further model, we went on constraining betas from the sequential working memory factor to each mathematic subdomain to be equal. The fit of this model, was similar to the previous model, *χ*^2^(57) = 61.88, *p* = 0.306, RMSEA = 0.020, SRMR = 0.035, CFI = 0.996, NNFI = 0.993, AIC = 13,194. Also in this case, this latter model had a lower AIC, was more parsimonious, and was not statistically different from the previous one, Δ*χ*^2^(5) = 7.85, *p* = 0.1645. These findings taken overall indicate that increasing the complexity of the model and assuming different betas (i.e., different relationships) between the simultaneous and sequential factor to each mathematic subdomain was not necessary.

**Fig. 2:**
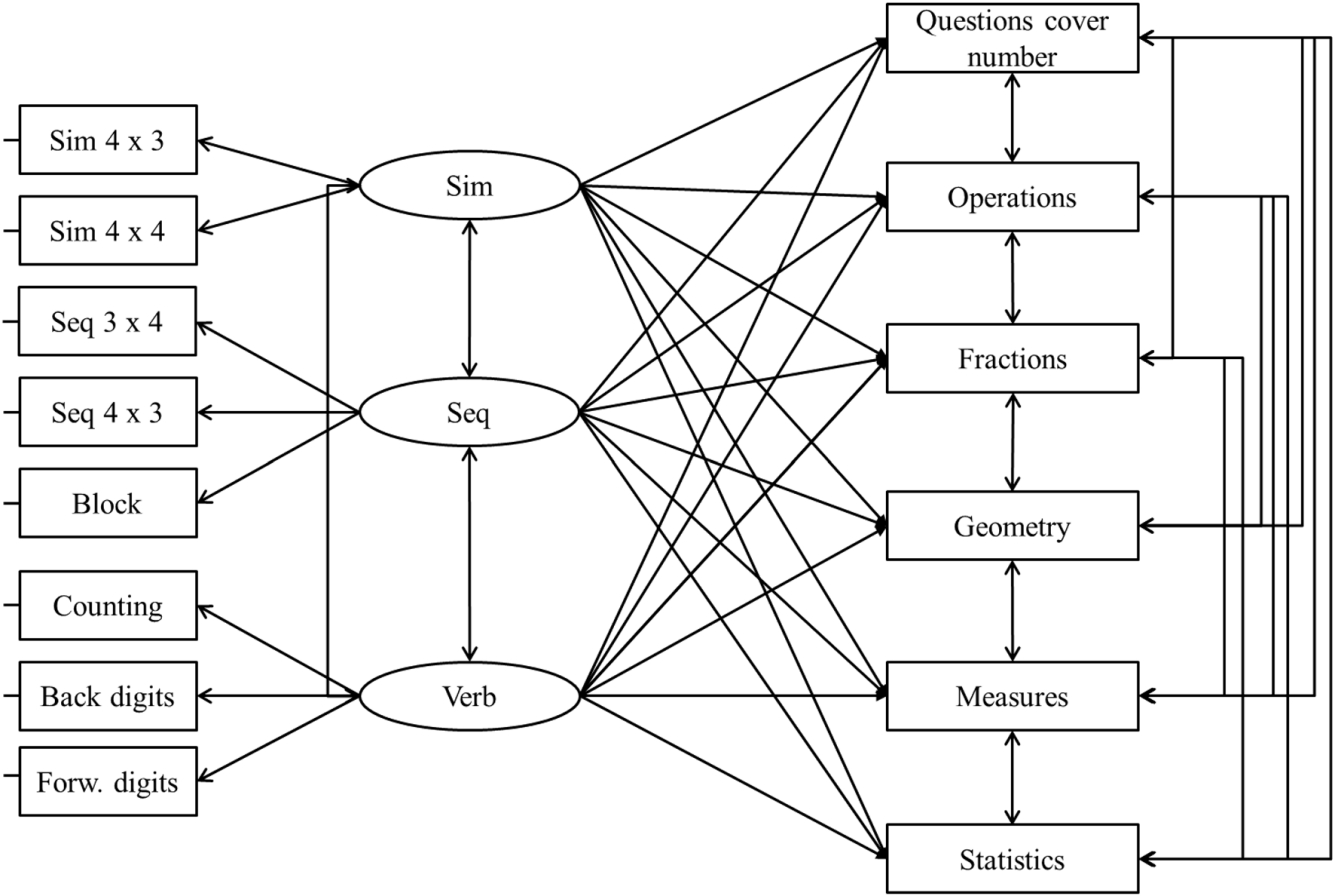
Theoretical model for the relationship between WM factors with observed mathematical subtests

In a further model, we went further on constraining the betas from the verbal working memory factor to each mathematic task. The fit of this model was somewhat poorer as compared to the previous one, *χ*^2^(62) = 89.36, *p* = 0.013, RMSEA = 0.046, SRMR = 0.081, CFI = 0.975, NNFI = 0.964, AIC = 13,211, Δ*χ*^2^(5) = 25.55, *p* = 0.0001. Such a finding indicates the possible presence of misfit, which was examined looking at modification indices and residuals. The inspection of the model led us to free one of the betas (i.e., the link from the verbal working memory factor to the operations component). This resulted in a considerably better fit, *χ*^2^(61) = 68.96, *p* = 0.226, RMSEA = 0.025, SRMR = 0.052, CFI = 0.993, NNFI = 0.989, AIC = 13,192. Comparing this model with all the previous ones we also established that this was the best fitting model as it had a lower AIC and was statistically superior as compared to all previous models. These findings taken together indicate that betas from simultaneous, sequential and verbal factors to each individual mathematic subdomain are similar, with only one exception (Fig. 2).

## Discussion

This study aimed to investigate the contributions to written mathematics made by verbal, spatial–simultaneous, and spatial–sequential working memory over the period of 2 years. Previous results (Allen et al., 2020b) highlighted a significant relationship between mathematics and spatial–simultaneous and verbal working memory in 7–8-year-old children. Therefore, we aimed to assess whether this relationship remained stable 2 years later or varied as a function of age.

From the correlations table, all correlations (both normal and after covarying for age) between mathematics, measured at T1 and T2, and working memory measures were statistically significant. This suggests that working memory is related to mathematics, both at T1 and T2. With regard to our specific research question for this paper, we identify a shift in the influence of the components of working memory on mathematics. Whilst verbal working memory remains a significant predictor, spatial–simultaneous becomes non-significant and is taken over by spatial–sequential. The relationship between spatial–sequential working memory and mathematics is stronger than that between verbal working memory and mathematics, though not significantly so, however, the strongest relationship remains between mathematics at T1 and T2. It was anticipated that this would be the case, therefore, the model was built in such a way that the significant relationships identified between spatial–sequential and verbal working memory and mathematics remain after accounting for the relationship between mathematics at T1 and T2. That is, these relationships reveal the amount of variance in mathematics that we are able to account for over and above that which is predicted by previous mathematical ability. As discussed in Allen et al. (2020b), the presence of a significant relationship with verbal working memory is supported by literature suggesting verbal-numeric tasks, as are those used in this study, show a direct relation to mathematical performance (as reviewed by Raghubar, Barnes, & Hecht, 2010).

There is a possibility that the influence of spatial–sequential working memory at T2 could be due to the complexity of the task. To complete sequential tasks, children are required to hold the initial elements of the stimuli sequence in mind for longer before recall, which could be considered more demanding than spatial–simultaneous tasks (Rudkin et al., 2007). This requirement to hold information for longer periods of time when encoded at different time points may replicate the child’s ability to handle sequentially derived information resulting from multi-step mathematics problems. Older children are more likely to encounter these types of problems in mathematics (Department for Education, 2013), therefore, spatial–sequential tasks may be more predictive of older children’s mathematical ability (Allen et al., 2020a), particularly if the proportion of multi-step versus single-step problems encountered also increases with age, as is often the case. As a result, it may be that spatial–sequential working memory is more strongly related to mathematics than verbal working memory due to the way the information is encoded. Similarly, Caviola, Colling, Mammarella and Szűcs (2020) suggest that spatial working memory may provide the mental workspace required to complete mathematics tasks, which is likely to be increasingly important in multi-step tasks.

Understanding the task demands of the working memory tasks themselves, particularly the spatial–sequential tasks, cannot be an influencing factor in their relationship with mathematics in this study, because working memory measures were taken at T1 only, as opposed to being repeated at T2. Only the mathematics measure was repeated at T2. This calls into question the evidence that high and low ability children in mathematics are not distinguishable by their spatial–sequential working memory (Bull, Johnston, & Roy, 1999), as the current result would suggest this may be possible. There is, however, an alternative argument by Andersson and Lyxell (2007), D’Amico and Guarnera (2005), McLean and Hitch (1999) that our current results support. Caution should be applied when trying to define distinct groups of children in mathematics based on their cognitive profile, as there is little evidence of a distinct profile of poor performers in mathematics in those without a diagnosis of mathematics difficulties.

Based on previous research suggesting a declarative shift (see Schneider, 2008 for a review of this literature), it is surprising that spatial–sequential working memory remains so influential in 9–10-year-old children. It has long been considered that younger children rely on using visuospatial working memory for mathematics (Van de Weijer-Bergsma et al., 2015), potentially because it acts as a mental ‘checker’ or allows them to use visual strategies that young children rely on so heavily. When children are first introduced to mathematical concepts, wherever possible the concept is made concrete through the use of tangible examples with blocks or counters, for example. This is done to give the children a concrete, visible reference point for the concept that they are able to interact with (e.g., draw on, rotate). Once they understand the material well enough, the scaffolding of concrete examples is slowly removed to make the work more abstract, using less tangible representation. By following this pattern, it is clear to see why the suggestion is made that children will rely more on visuospatial working memory in their younger years, before making the transition to using verbal working memory resources when they are older. However, the group of children used in this study are older than the age at which this declarative shift is predicted to take place (around 7 years of age, Schneider, 2008), therefore, suggesting that a shift of this nature may not tell the whole story.

One potential explanation relates to the relative lack of evidence regarding the individual contributions of the subtypes of visuospatial working memory to mathematical performance. Although not a definitive claim, a meta-analysis by Allen, Higgins and Adams (2019) suggests some influence of the type of visuospatial working memory measured on the magnitude of the effect size measured in studies relating to mathematics. This synthesis identified that the relationship between spatial–sequential working memory and mathematical reasoning (problem solving; a large portion of the mathematics test used in this study) had not previously been investigated. As such, this paper may go some way to shedding light on this relationship, highlighting a lack of a thorough understanding of the interplay between mathematics and the subtypes of visuospatial working memory previously. This is notable, because the involvement of elements of visuospatial working memory in older children is supportive of other recent findings (Allen et al., 2020a). Unlike previous work suggesting a fundamental shift in the reliance on components of working memory for mathematics, the results of this study, taken as a whole, suggest verbal working memory makes a relatively stable contribution to performance, with the variability emerging from the involvement of the components of visuospatial working memory, shifting from simultaneous to sequential influence (see Allen et al., 2020b for further information on T1 of this study).

It is unlikely, though not impossible, that the shift we see in the involvement of working memory is due to the cognitive load imposed by the task as tasks are always visible and children have the opportunity to write down any workings or intermediate results, and so are not required to hold these items in mind. However, there is the possibility that children, particularly those who are anxious for example, may face more difficulties under timed conditions (Ashcraft & Moore, 2009; Carey et al., 2016; Onwuegbuzie & Seaman, 1995). There is also some evidence that children who have poor working memory are also poor at comprehending text (e.g., Carretti, Cornoldi, De Beni, & Palladino, 2004). Similarly, task instructions are always present meaning children have the opportunity to break tasks down into smaller chunks, though those with particularly poor working memory may have difficulties with keeping their place in the instructions (Alloway, 2006; Gathercole & Alloway, 2004). Due to the nature of the paper layout, extraneous cognitive load is relatively low as information is presented alongside the associated question and graphs and diagrams are interspersed through the text in the most appropriate place. There may be some influence of cognitive load due to the increased number of multi-step questions designed for older children requiring the maintenance of intermediate steps (Sweller, 1994), but this should be minimal in this case and is unlikely to fully explain the results found.

The proportions of questions concerning the different domains of mathematics could potentially influence the results over time, even though children completed the same longitudinal test (albeit the opposite paper at T2, balanced exactly for difficulty and weightings towards the different domains). All of the questions were included on the paper at T1, and some children made attempts at these, however, children will have been able to access a greater number of these questions at T2 following 2 years of extra schooling. There is no evidence from a visual search of the frequency of questions relating to each question type that this changes over the course of the test. If this were the case, it may be that working memory influence shifts as a direct consequence of more questions being asked that tap different working memory components later in the paper, thus only older children will be able to access them. This is not the case. As such, it follows that, when developing a screening measure, children should be screened on measures that are predictive over longer periods of time. It is important to include shorter term predictors of mathematics as well to pick up children who are likely to fall behind immediately, however, the focus should be on longer term predictors.

As with T1 of this study, there are some inherent limitations. Primarily, the use of a verbal-numeric measure of verbal working memory. Verbal-numeric working memory has been shown to demonstrate a different relationship to mathematics than verbal working memory measures using stimuli not relating to numbers (see Raghubar et al., 2010 for a review of this literature). After highlighting this as an issue at T1, Allen et al. (2020a) found a similar pattern of results using non-numeric verbal stimuli. The inclusion of only typically developing children has not, however, been addressed at this time as a clear understanding of typical development is necessary before investigating the nature of the relationship in atypical samples, such as those with diagnosed mathematics difficulties. As a result, we, therefore, remain unable to compare the development of typical and atypical populations to assess any differences.

In this paper we have also attempted to distinguish between different mathematics domains at Y5. Intriguingly, the relationship between simultaneous and sequential working memory factors with the different mathematic subdomains seems to be quite similar. A recent meta-analysis by Peng et al. (2016) tested the relationship between working memory and different mathematics domains, demonstrating some small variations in terms of the correlations between mathematic subdomains (from 0.23 to 0.37). In fact, one could expect, for example, geometry to draw more on visuospatial skills. However, geometry seems to be a very complex domain involving several complex abilities (Mammarella et al., 2017). One possibility is that our results at Y5 are influenced by the nature of the geometry tasks at this stage in the curriculum. In a similar study, Giofrè, Mammarella and Cornoldi (2014), with a sample of 4th and 5th graders, found that working memory, independent of the modality, had the highest correlation with geometry. This finding was explained by the authors arguing that formal education in geometry, at this stage, involves both visuospatial and verbal materials (such as texts, definitions, formulae, and theorems). Therefore, the absence of the stronger influence of visuospatial working memory is not necessarily surprising. As for the verbal working memory factor, the pattern was rather similar but with one exception.

Results reported in the present paper show that verbal working memory has the explanatory power in all mathematics domains. Intriguingly, the link between verbal working memory and a specific component (i.e., operations) seemed to be higher as compared with the other tasks. It could be argued that the manipulation of operations could draw on verbal and visuospatial working memory to a large extent (Caviola et al., 2012; Van de Weijer-Bergsma et al., 2015).

Future research should seek to continue to address the limitations presented here, as well as to build upon the findings presented to continue to develop our understanding of the relationships between the components and subcomponents of working memory and mathematics. Once this understanding has been developed, researchers can begin to work with atypical populations to try to ascertain whether these populations differ from typical populations in the ways in which working memory contributes to task completion. There are clear implications for education providers and researchers as in developing our understanding of this area, we will be able to use this knowledge to support children who have difficulties in mathematics through supporting their working memory. By understanding which elements of working memory are most important for mathematics at different ages, educators will be able to provide targeted support for children in the form of aids and alternative methods where necessary.

In conclusion, this study confirmed that it is possible to predict mathematics using working memory data gathered 2 years previously, however, that the specific nature of the relationship changes over time. Spatial–sequential and verbal working memory tasks are predictive of 9–10-year-old performance in mathematics, as opposed to spatial–simultaneous and verbal measures in the same children at 7–8 years of age.
